# Photocurrent enhancements of organic solar cells by altering dewetting of plasmonic Ag nanoparticles

**DOI:** 10.1038/srep14250

**Published:** 2015-09-21

**Authors:** Tyler Fleetham, Jea-Young Choi, Hyung Woo Choi, Terry Alford, Doo Seok Jeong, Taek Sung Lee, Wook Seong Lee, Kyeong-Seok Lee, Jian Li, Inho Kim

**Affiliations:** 1 School for Engineering of Matter, Transport and Energy, Arizona State University, Tempe, Arizona, 85287 USA; 2 Electronic Materials Research Center, Korea Institute of Science and Technology, Hwarangno 14 gil-5 Seongbuk-gu Seoul 136-791 Republic of Korea

## Abstract

Incorporation of metal nanoparticles into active layers of organic solar cells is one of the promising light trapping approaches. The size of metal nanoparticles is one of key factors to strong light trapping, and the size of thermally evaporated metal nanoparticles can be tuned by either post heat treatment or surface modification of substrates. We deposited Ag nanoparticles on ITO by varying nominal thicknesses, and post annealing was carried out to increase their size in radius. PEDOT:PSS was employed onto the ITO substrates as a buffer layer to alter the dewetting behavior of Ag nanoparticles. The size of Ag nanoparticles on PEDOT:PSS were dramatically increased by more than three times compared to those on the ITO substrates. Organic solar cells were fabricated on the ITO and PEDOT:PSS coated ITO substrates with incorporation of those Ag nanoparticles, and their performances were compared. The photocurrents of the cells with the active layers on PEDOT:PSS with an optimal choice of the Ag nanoparticles were greatly enhanced whereas the Ag nanoparticles on the ITO substrates did not lead to the photocurrent enhancements. The origin of the photocurrent enhancements with introducing the Ag nanoparticles on PEDOT:PSS are discussed.

Organic solar cells (OSCs) based on conducting polymers and small molecular weight organic semiconductors have attracted much research interest owing to their ease of fabrication at low temperature, low material cost and inherent flexibility of organic materials^1^,^2^,^3^. Intensive research has been carried out over the last decade to improve the power conversion efficiency of OSCs through the development of novel materials and effective device engineering. The power conversion efficiency of OSCs has steadily increased and has exceeded 10% recently^4^,^5^. However, the efficiency must be improved further in order to make OSCs commercially viable. Effective light harvesting in solar cells is one of the fundamental requirements to realize high efficiencies. The straightforward scheme of increasing active layer thickness to harness more photons is limited by charge carrier diffusion lengths which constrain OSC active layers to thicknesses ranging from 100 nm to 200 nm^6^,^7^. Although most organic semiconductors, particularly donor materials, are strong optical absorbers, thicknesses in this range are not sufficient to fully harvest incident photons.^8^ Thus, effective light trapping is necessary to provide a route to further improvements of power conversion efficiencies of OSCs.

Plasmonic light trapping utilizing metallic nanoparticles such as Ag and Au is one promising approach to photocurrent enhancement^9^,^10^,^11^. Metal nanoparticles can be easily prepared by chemical synthesis or physical vacuum deposition^12^,^13^,^14^. Metal nanoparticles are typically embedded into active or buffer layers, or they can be placed at the interfaces of active and buffer layers^15^. Localized surface plasmon resonance (LSPR) arising from collective oscillations of free electrons can be excited at the metal nanoparticles with incident photons having a resonance frequency^16^. Excitation of LSPR leads to amplified optical fields by approximately a factor of two orders of magnitude at the interface of metal nanoparticles^17^. Incident photons into the metal nanoparticles are either absorbed in the metal nanoparticles by heat dissipation or scattered into the medium. If the medium is strongly absorbing as is the case for donor materials in OSCs, then some of the amplified light fields, which are otherwise dissipated as heat in the metal nanoparticles, would transfer and be absorbed by the absorbing medium^18^,^19^. Nevertheless, parasitic absorption in the nanoparticles is still present. On the other hand, preferential scattering by the metal nanoparticles is desirable in solar cell applications because the light scattered into the medium could be harvested and converted into electricity. In general, by increasing the diameter of the metal nanoparticles a larger proportion of the light can be scattered by spherical metal nanoparticles rather than be absorbed^17^. In most research work, the metal nanoparticles of the diameter ranging from 10 nm to 80 nm were applied in a colloidal form^15^.

In this study, we physically synthesize Ag nanoparticles by thermal evaporation and perform post-annealing to induce enlarging of the metal nanoparticles by dewetting of the evaporated metal nanoparticles. The shape of the Ag nanoparticles on indium-tin-oxide (ITO) glass changes slightly with post-annealing, and their size is simply controlled by varying their nominal thickness. More interestingly, the size of the Ag nanoparticles is greatly enlarged with employing a polymer buffer layer, poly(3,4-ethylenedioxythiophene):poly(styrenesulfonate) (PEDOT:PSS) on ITO glass. The substantial enlargements of the Ag nanoparticles lead to plasmonic absorption enhancements in the active layers, resulting in enhanced photocurrents. We experimentally demonstrate that photocurrents of the OSCs can be enhanced by over 30% with incorporation of Ag nanoparticles on the buffer layers. We also demonstrate that the Ag nanoparticles prepared on PEDOT:PSS coated ITO glass provide remarkable enhancements by a factor of 2.1 in external quantum efficiency (EQE) of the solar cells compared to the control device without Ag nanoparticles. Using the Mie theory and the finite-difference-time-domain simulations, we identified that the EQE enhancements in the solar cells arise from the enhanced scattering by the localized surface plasmon resonances of the Ag nanoparticles.

## Results and Discussions

**Figure 1:**
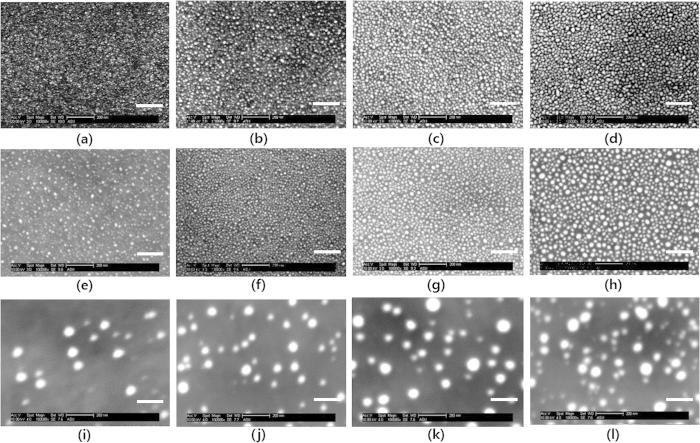
SEM images of the Ag nanoparticles on different substrates. The as-deposited Ag nanoparticles on ITO glass for the nominal thicknesses of (**a**) 1 nm, (**b**) 2 nm, (**c**) 3 nm, and (**d**) 4 nm. The annealed Ag nanoparticles on ITO glass for the nominal thicknesses of (**e**) 1 nm, (**f**) 2 nm, (**g**) 3 nm, and (**h**) 4 nm. The annealed Ag nanoparticles on PEDOT:PSS coated ITO glass for the nominal thicknesses of (**i**) 1 nm, (**j**) 2 nm, (**k**) 3 nm, (**l**) 4 nm. The white scale bars in each picture denotes 200 nm.

**Figure 2:**
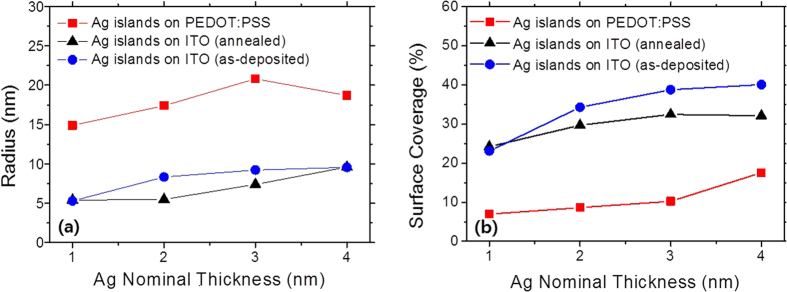
(**a**) The radius and (**b**) surface coverage of the Ag nanoparticles prepared on ITO glass and PEDOT:PSS coated ITO glass with varying the nominal thicknesses and annealing.

Ag nanoparticles were physically synthesized by a thermal evaporator on ITO substrates with varying their nominal thicknesses from 1 nm to 4 nm. The substrate was not intentionally heated and kept at room temperature. The as-deposited Ag nanoparticles were subsequently annealed in vacuum at 300 °C for 30 min to enlarge their size. We introduced a conducting polymer of PEDOT:PSS as a buffer layer to modify the surface adhesive force of the substrate to promote dewetting of the Ag nanoparticles^20^. In the same manner, we deposited the Ag nanoparticles on PEDOT:PSS with varying the nominal thicknesses from 1 nm to 4 nm and annealed them at 200 °C for 10 min. PEDOT:PSS is thermally degraded at higher temperature than 250 °C, which is the reason we chose lower annealing temperature^21^. The SEM images of the Ag nanoparticles on each substrate were shown in Fig. 1(a)–(l). The size and coverage of the Ag nanoparticles were tuned by simply varying their nominal thicknesses and performing annealing. Statistical analyses on the sizes and coverages of the Ag nanoparticles reveal the distinct differences of nanoparticle forming behaviors depending on substrates as shown in Fig. 2(a,b). The sizes of Ag nanoparticles on ITO substrates were slightly enlarged with increasing their nominal thicknesses. Furthermore, the as-deposited Ag nanoparticles on ITO substrate became slightly larger and more spherical with annealing. On the other hand, the surface coverages of the Ag nanoparticles on the ITO substrate were decreased slightly with annealing. Interestingly, the Ag nanoparticles were almost three times larger with introducing the buffer layer of PEDOT:PSS and annealing. In turn, the surface coverages of the Ag nanoparticles on PEDOT:PSS were proportionally decreased.

**Figure 3:**
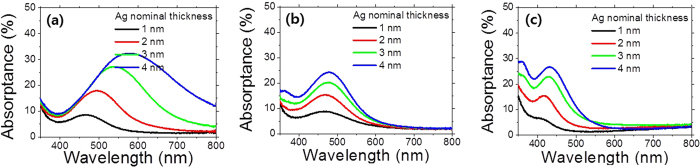
(**a**) The optical absorptances of the as-deposited Ag nanoparticles on ITO glass. The optical absorptances of the annealed Ag nanoparticles for different substrates of (**b**) ITO glass and (**c**) PEDOT:PSS coated ITO glass, respectively.

The characteristic absorption of the Ag nanoparticles arising from the localized surface plasmon resonances (LSPR) were measured using a UV-Vis spectrophotometer *and shown in* Fig. 3. It is well known that the LSPR frequency of plasmonic metal nanoparticles is sensitive to its shape and size^16^,^17^,^18^,^22^. The peak absorption wavelengths of the as-deposited Ag nanoparticles shift toward the longer wavelengths noticeably with increasing their nominal thicknesses. The peak absorption wavelengths increase from 467 nm up to 575 nm with varying the nominal thicknesses of the Ag nanoparticles from 1 nm to 4 nm. This is because the shape of the Ag nanoparticles becomes more elongated with increasing their nominal thicknesses as has been previously reported in literature^23^. After annealing the Ag nanoparticles on ITO substrate, the peak absorption greatly shift toward the shorter wavelengths around 470 nm. As shown in the SEM images of the Ag nanoparticles, the shape of the Ag nanoparticles become more spherical with annealing, resulting in the blue-shifts of the peak absorption wavelengths. The plasmonic resonance wavelength is sensitive to the shape of the Ag nanoparticles^16^. As the Ag nanoparticles are elongated more laterally, the resonance wavelengths red-shift. With annealing the Ag nanoparticles, they become more spherical, resulting in the blue-shifted resonance wavelengths. As for the Ag nanoparticles prepared on PEDOT:PSS coated ITO substrate, the peak absorption wavelengths even further shift toward the shorter wavelengths. The buffer layer of PEDOT:PSS reduces the surface adhesive force of ITO substrate; this appears to promote Ag nanoparticle dewetting, resulting in dramatic size enlargements as seen in the SEM images^24^. The greater size of plasmonic metal nanoparticles is more beneficial for predominant scattering over absorption by the LSPR; thus, introduction of the buffer layer, PEDOT:PSS would be more preferable.

**Figure 4:**
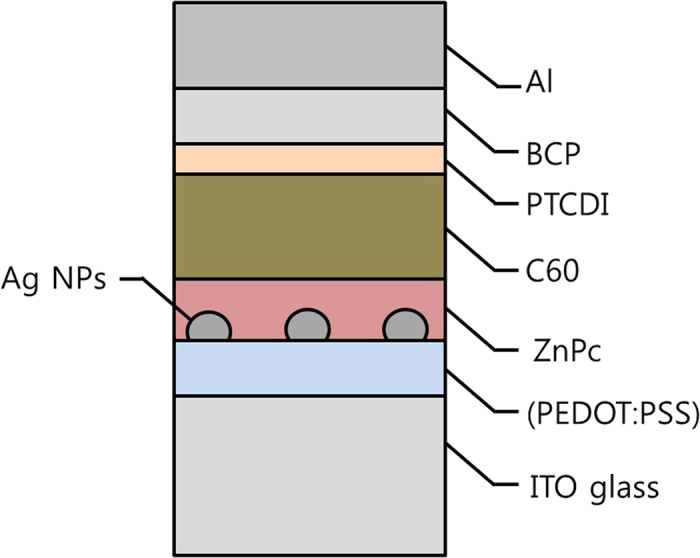
The schematic of device structure of ZnPc/C_60_ organic solar cells with incorporation of the Ag nanoparticles on ITO or PEDOT:PSS coated ITO glass.

**Figure 5:**
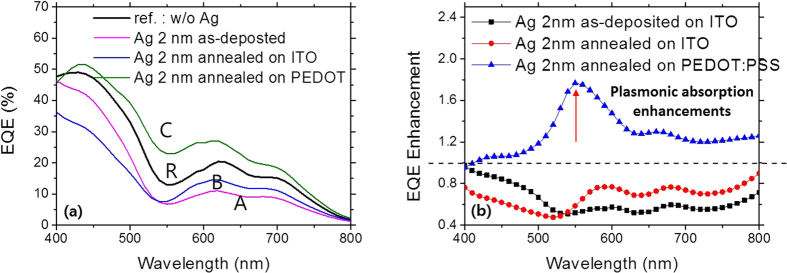
(**a**) The EQE spectra of the devices without the Ag nanoparticles and with the annealed Ag nanoparticles on the different substrates. (R) denotes the reference device without the Ag nanoparticle, (A) the device with the as-deposited Ag nanoparticles of 2 nm nominal thickness, (B) the device with the annealed Ag nanoparticles on ITO, and (C) the device with the annealed Ag nanoparticles on PEDOT:PSS. (**b**) The EQE enhancements of the devices with the as-deposited Ag nanoparticles and with the annealed ones for the different substrates of ITO glass and PEDOT:PSS coated ITO glass. The red arrow denotes a peak EQE enhancement.

Organic solar cells based on small molecular weight organic semiconductors were fabricated and the plasmonic Ag nanoparticles were exploited in order to enhance optical absorption of thin active layers. The device schematic with incorporation of the Ag nanoparticles was illustrated in Fig. 4. Two types of device structures were fabricated as follows depending on inclusion of the buffer layer: Structure 1: ITO glass/ZnPc 10 nm/C_60_ 30 nm/PTCDI-C6 10 nm/BCP 14 nm/Al 100 nm and Structure 2: ITO glass/PEDOT:PSS 40 nm/ZnPc 10 nm/C60 30 nm/PTCDI-C6 10 nm/BCP 14 nm/Al 100 nm. The Ag nanoparticles were inserted between ITO glass and ZnPc or between PEDOT:PSS and ZnPc. The presence of PEDOT:PSS in our organic solar cells (Structure 2) has minimal influence on the device parameters and only modify the surface adhesive force of ITO glass *as is shown in* Table 1^25^. We prepared four different solar cells for comparative study of the effects of the Ag nanoparticle size: (R) the reference device without the Ag nanoparticles of Structure 1, (A) the device with the as-deposited Ag nanoparticles of Structure 1, (B) the device with the annealed Ag nanoparticles of device structure 1, (C) the device with the annealed Ag nanoparticles of Structure 2. The Ag nanoparticles were deposited by evaporation of 2 nm nominal thickness Ag. The EQEs of four cells were measured in the wavelength range of 400 nm ~ 800 nm and shown in Fig. 5(a). Note that only device (C) has enhanced the EQE values in almost entire spectral ranges while device (A) and (B) have the reduced EQE values compared with the reference, device (R). Figure 5(b) shows the EQE enhancements of three cells (A), (B), and (C) which are normalized to the reference device (R). Device (C) has a characteristic peak around the wavelength of 550 nm. The maximum EQE enhancement value of device (C) was ~1.8 at the wavelength of 550 nm. This is a very interesting result because main differences from those three devices (A, B, C) are size of the Ag nanoparticles. From the Mie theory, it is well known that scattering efficiency of a nanoparticle is greater as the nanoparticle size is larger^17^,^26^. The optical field amplification by the LSPR of plasmonic metal nanoparticles is always accompanied by parasitic absorption losses of metal nanoparticle itself. Because the parasitic absorption loss in the metal nanoparticle contrary to the scattering efficiency does not contribute to the photo-generation, it needs to be minimized if possible. The scattering efficiency scales more rapidly with increasing the size of metal nanoparticles compared with the absorption efficiency; thus, the use of larger metal nanoparticles is desirable^16^. The enlargements of the Ag nanoparticles with introduction of the buffer layer, PEDOT:PSS in device (C) would lead to enhanced scattering, resulting in improved EQEs of the device.

**Figure 6:**
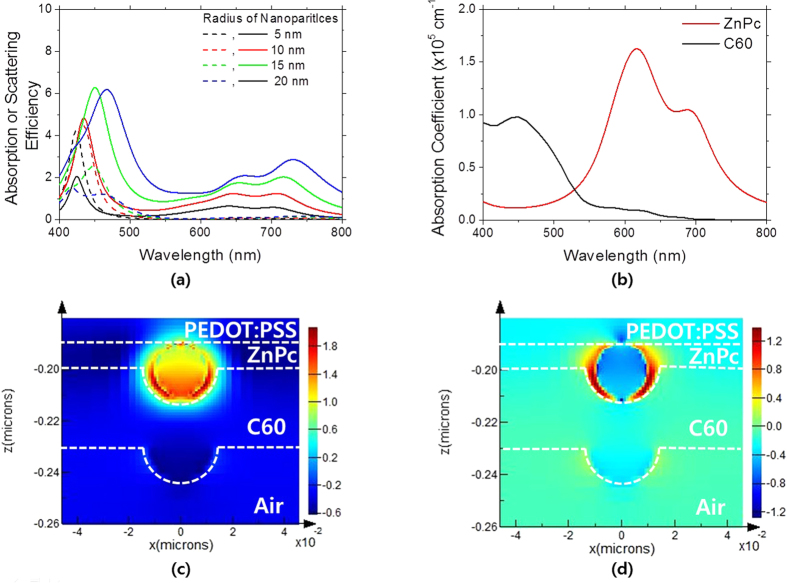
(**a**) The calculated absorption (*dashed lines*) and scattering (*solid lines*) efficiencies of a spherical Ag nanoparticle embedded in ZnPc for various sizes using the modified Mie theory. (**b**) The absorption coefficients of ZnPc and C_60_, which were extracted from the refractive indices determined by spectroscopic ellipsometry (J.A. Woollam M-2000). (**c**) The simulated *E*-field intensity profiles for ZnPc/C60 bilayers with incorporation of the Ag nanoparticles on PEDOT:PSS coated ITO glass for the resonance wavelength, 424 nm and (**d**) the off-resonance wavelength, 550 nm. For the FDTD calculations, the incident light was assumed to be polarized in the x-direction.

**Figure 7:**
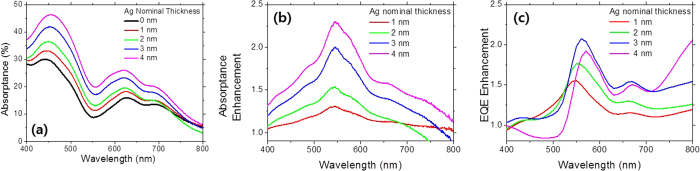
(**a**) The absorptance spectra and (**b**) the absorptance enhancements of the ZnPc/C_60_ bilayers with the Ag nanoparticles on PEDOT:PSS coated ITO glass for the various Ag nominal thicknesses. (**c**) The EQE enhancements of the devices with incorporation of the Ag nanoparticles into the interface of PEDOT:PSS/ZnPc.

The standard Mie theory was developed to understand light scattering from a nanoparticle in a non-absorbing media. But in this study, metal nanoparticles are embedded in an absorbing medium which are active layers of organic solar cells. The standard Mie theory needs to be modified to analyze light scattering from a nanoparticle in an absorbing medium, and some literature has been published to address this issue. Using the modified Mie theory developed in the previous literature^19^, we calculated the scattering and absorption efficiencies of the Ag nanoparticle in a donor material of ZnPc in our organic solar cells with varying the size of the Ag nanoparticle, which are plotted in Fig. 6(a). The size of the Ag nanoparticles was varied from a radius 5 nm to 20 nm. The absorption efficiency of the Ag nanoparticles has strong spectral responses in narrow wavelength range of 400 nm ~ 500 nm while the scattering efficiency has strong peaks in the similar ranges and also strong ones in the longer wavelength range of 600 nm ~ 750 nm. As shown in Fig. 6(b), ZnPc has strong absorption in the wavelength range of 600 nm ~ 750 nm. The LSPR wavelengths of the Ag nanoparticles, as seen in their absorption efficiency spectra, lie around in the wavelength range of 400 nm ~ 500 nm. The scattering efficiency spectrum is spectrally broader compared with that of the absorption efficiency for all the Ag nanoparticles. This is because the absorbing medium partially absorbs the amplified evanescent fields from the Ag nanoparticles by the LSPR effect depending on the absorption strength of the medium^19^. All the photon energy transferred into the medium is counted as scattered one in our calculation; thus, scattering is also strong in the wavelength ranges where the absorption coefficients are high. Furthermore, similarly to the case of a nanoparticle in a non-absorbing medium, the scattering efficiency becomes more dominant over the absorption efficiency as the size of the Ag nanoparticles becomes larger. Especially when the size of the Ag nanoparticles is greater than 20 nm, the scattering efficiency is greater than the absorption efficiency in our calculations. In this regards, the Ag nanoparticle greater than 20 nm is more preferable for absorption enhancements (Fig. 7).

The electric field (*E*-field) intensity profiles near the Ag nanoparticles of a 10 nm radius were calculated using the FDTD simulations. We assumed that a spherical Ag nanoparticle is placed on PEDOT:PSS, and the ZnPc, C_60_ layers are conformally coated on the Ag nanoparticle as shown in Fig. 6(c,d). On the resonance wavelength, the *E*-field intensity near the nanoparticle as well as inside the nanoparticle is greatly amplified. The intensified *E*-field inside the nanoparticle would lead to the parasitic absorption. On the other hand, on the off-resonance wavelength of 550 nm, the *E*-field intensity inside the Ag nanoparticle is very weak as seen in Fig. 6(d) while that near the Ag nanoparticle is strong. This explains well the reason the absorption efficiency on the off-resonance wavelengths is negligible^27^.

The annealed Ag nanoparticle on PEDOT:PSS has a diameter of 17.4 nm. The experimental EQE enhancement peak lies in the wavelength of ~550 nm as shown in Fig. 5(b). The simulated peak scattering efficiency of the 20 nm diameter Ag nanoparticle in ZnPc lies on the wavelength of ~435 nm, which is shorter than the experimentally measured peak wavelength. This disagreement appears to arise from our assumption of the spherical shape of the Ag nanoparticles. The red-shifted LSPR wavelengths imply that the shape of the Ag nanoparticles would be elongated.

We deposited the Ag nanoparticles on PEDOT:PSS coated ITO glass by varying the nominal thickness, and sequentially evaporated ZnPc and C_60_. We measured optical absorptions for those samples and plotted them with the reference sample without the Ag nanoparticles in Fig. 7. As expected, the optical absorptions increased consistently with increasing the Ag nominal thickness. The absorption enhancements normalized to the reference sample showed peak absorptions around 550 nm, which is attributed to the LSPR effect. In the similar wavelength range, the devices containing the Ag nanoparticles showed the peak EQE values as seen in Fig. 7(c). The device with the 3 nm Ag nominal thickness showed the maximum EQE enhancement of 2.1. The device with the 4 nm Ag nominal thickness showed slightly lower EQE values and greatly reduced EQE values in short wavelength ranges below 500 nm were observed. As shown in Fig. 2, the size of the Ag nanoparticles reach the maximum value at the 3 nm nominal thickness and becomes slightly reduced with further increase in the nominal thickness while the surface coverage increases consistently. This can be understood by following explanations. The increased coverages result in the increased parasitic absorption losses; thus, without further increase in the size of Ag nanoparticles like the case of the device with the 4 nm Ag nominal thickness, the increased coverages would lead to the increased parasitic absorption. Furthermore, the Ag nanoparticles located on the front surface of the organic solar cells reduce the absorption in C_60_ by blocking the incident light; thus, the device with the 4 nm Ag nanoparticles has reduced the EQE values in the short wavelengths below 500 nm.

Table 1 shows the device parameters for the solar cells we made in this study. All the devices were tested under illumination of AM(air mass) 1.5 G at a light intensity of 100 mW/cm^2^. The cells without the Ag nanoparticles have the photocurrents of 5.91 mA/cm^2^ and 5.88 mA/cm^2^ for ITO and ITO/PEDOT:PSS anodes, respectively. The cells with the Ag nanoparticles on ITO do not have improved photocurrents, whereas the cells with the Ag nanoparticles on PEDOT:PSS have enhanced photocurrents ranging from 6.85 mA/cm^2^ to 7.72 mA/cm^2^. Although the devices have enhanced photocurrents with the Ag nanoparticles on PEDOT:PSS, the efficiency is not higher than the cells without the Ag nanoparticles. This is because *V*_*oc*_ and *FF* of those devices are poor. One of the main reasons for poor *V*_*oc*_ would be a low work function of Ag (~4.3 eV)^28^. Passivation of the Ag nanoparticles with higher work function materials or insulating materials would be one way to keep *V*_oc_ with enhanced photocurrents, which would lead to enhanced power conversion efficiency.

In conclusion, we prepared the Ag nanoparticles by thermal evaporation and subsequent post annealing on ITO glass and PEDOT:PSS coated ITO glass, respectively. By simply varying the nominal thickness of Ag, the coverage and size of the Ag nanoparticles were able to be tuned. The size of the Ag nanoparticles on PEDOT:PSS was more than three times of those on ITO glass, which is attributed to the lower surface adhesive force of the PEDOT:PSS coated ITO glass. We fabricated organic solar cells by stacking organic semiconductor layers sequentially onto ITO glass and PEDOT:PSS coated ITO glass with the Ag nanoparticles. The enlarged Ag nanoparticles by introduction of the buffer layer, PEDOT:PSS enhance light scattering over parasitic absorption, leading to enhanced photo-generation of organic solar cells near the plasmonic Ag nanoparticles. With the optimal size of the Ag nanoparticles, the maximum EQE was enhanced by a factor of 2.1, and the photocurrent was increased by 30% with reference to the device without the Ag nanoparticles. Altering the dewetting behavior of the metal nanoparticles by introduction of buffer layers we proposed in this study would be a simple and promising route to enhance photocurrents of thin organic solar cells.

## Methods

For the fabrication of plasmonic Ag nanoparticles, Ag was deposited on clean ITO glass by thermal evaporation at an evaporation rate of 0.2 Ǻ/sec, which was monitored using a quartz crystal monitor. The Ag nominal thicknesses were adjusted from 1 nm to 4 nm. Subsequently, as-deposited Ag nanoparticles were annealed in N_2_ atmosphere at 300 °C for 30 min. As for the case with the buffer layers, PEDOT:PSS was spin-coated onto ITO glass at the spinning rate of 4,000 rpm and baked at 190 °C for 10 min. The size and coverage of Ag nanoparticles were determined by scanning electron microscopy (SEM), and statistical analyses with image processing using commercial software (Image-Pro Plus) were carried out. Optical absorptances of plasmonic Ag nanoparticles were measured by determining of their reflectances and transmittances using an integrating sphere of a spectrophotometer (Cary 5000). For organic solar cell fabrication, zinc phthalocyanine (ZnPc) and fullerene (C_60_) were thermally evaporated onto ITO glass and PEDOT:PSS coated ITO glass as donor and acceptor. Subsequently N,N’-dihexyl-3,4,9,10-perylenedicarboximide (PTCDI-C6) and bathocurpoine (BCP) were deposited as buffer layers, and devices were finalized with aluminum deposition as cathode. C_60_ and BCP were supplied by MER Corporation and Sigma Aldrich, respectively, while ZnPc and PTCDI-C6 were synthesized in house following previous literature^29^,^30^. For plasmonic absorption enhancements of the organic solar cells, Ag nanoparticles of various sizes and coverages were employed on ITO glass and PEDOT:PSS coated ITO glass. Current-voltage characteristics of the organic solar cells under a solar simulator with illumination of AM 1.5G at a light intensity of 100 mW/cm^2^ were also measured for extraction of solar cell parameters. EQE was measured at the wavelength range of 400 ~ 800 nm using a custom-setup (Optoronic Laboratories). The absorption and scattering efficiencies from a single Ag nanoparticle embedded in the donor material were calculated using the modified Mie scattering theory. Finite-difference-time-domain (FDTD) simulations in three dimensions were performed to identify the absorption enhancements with incorporation of the Ag nanoparticles in the organic solar cells using a commercial software package (Lumerical FDTD solutions). The periodic boundary conditions in x- and y-directions and perfectly matched layer (PML) conditions in z-direction were used.

## Additional Information

**How to cite this article**: Fleetham, T. *et al.* Photocurrent enhancements of organic solar cells by altering dewetting of plasmonic Ag nanoparticles. *Sci. Rep.* **5**, 14250; doi: 10.1038/srep14250 (2015).

## Figures and Tables

**Table 1:** The solar cell parameters of the devices with the Ag nanoparticles with various anode contacts.

**Anode contact**	**J_sc_(mA/cm^2^)**	**V_oc_(V)**	**FF**	**PCE(%)**
*ITO*	5.88	0.63	0.64	2.36
*ITO/PEDOT:PSS*	5.91	0.63	0.68	2.52
ITO/Ag 1 nm (as-dep.)	4.26	0.60	0.65	1.67
ITO/Ag 2 nm (as-dep.)	4.11	0.55	0.54	1.21
ITO/Ag 3 nm (as-dep.)	3.89	0.54	0.67	1.41
ITO/Ag 4 nm (as-dep.)	4.10	0.54	0.66	1.47
ITO/Ag 1 nm (annealed.)	4.33	0.57	0.67	1.67
ITO/Ag 2 nm (annealed.)	3.82	0.52	0.68	1.35
ITO/Ag 3 nm (annealed.)	3.60	0.44	0.64	1.01
ITO/PEDOT:PSS/Ag 1 nm (annealed.)	6.85	0.33	0.56	1.27
ITO/PEDOT:PSS/Ag 2 nm (annealed.)	7.17	0.23	0.49	0.81
ITO/PEDOT:PSS/Ag 3 nm (annealed.)	7.72	0.23	0.46	0.82
ITO/PEDOT:PSS/Ag 4 nm (annealed.)	6.97	0.28	0.52	1.01

The multilayers of ZnPc 10 nm/C_60_ 30 nm/PTCDI-C6 10 nm/BCP 14 nm/Al 100 nm were stacked onto the following anode contacts for the cell fabrications.
